# Immunotherapy Responsiveness and Risk of Relapse in Down Syndrome Regression Disorder

**DOI:** 10.21203/rs.3.rs-2521595/v1

**Published:** 2023-02-13

**Authors:** Jonathan Santoro, Noemi Spinazzi, Robyn Filipink, Panteha Hayati-Rezvan, Ryan Kammeyer, Lina Patel, Elise Sannar, Luke Dwyer, Abhik Banerjee, Mellad Khoshnood, Sabaj Jafarpour, Natalie Boyd, Rebecca Partridge, Grace Gombolay, Alison Christy, Diego Real de Asua, Maria del Carmen Ortega, Melanie Manning, Heather Van Mater, Gordon Worley, Cathy Franklin, Maria Stanley, Ruth Brown, George Capone, Elieen Quinn, Michael Rafii

**Affiliations:** Children’s Hospital Los Angeles; Kennedy-Krieger Institute and Johns Hopkins University; Kennedy-Krieger Institute and Johns Hopkins University; Kennedy-Krieger Institute and Johns Hopkins University; Kennedy-Krieger Institute and Johns Hopkins University; Kennedy-Krieger Institute and Johns Hopkins University; Kennedy-Krieger Institute and Johns Hopkins University; Kennedy-Krieger Institute and Johns Hopkins University; Kennedy-Krieger Institute and Johns Hopkins University; Kennedy-Krieger Institute and Johns Hopkins University; Kennedy-Krieger Institute and Johns Hopkins University; Kennedy-Krieger Institute and Johns Hopkins University; Kennedy-Krieger Institute and Johns Hopkins University; Kennedy-Krieger Institute and Johns Hopkins University; Kennedy-Krieger Institute and Johns Hopkins University; Kennedy-Krieger Institute and Johns Hopkins University; Kennedy-Krieger Institute and Johns Hopkins University; Kennedy-Krieger Institute and Johns Hopkins University; Kennedy-Krieger Institute and Johns Hopkins University; Kennedy-Krieger Institute and Johns Hopkins University; Kennedy-Krieger Institute and Johns Hopkins University; Kennedy-Krieger Institute and Johns Hopkins University; Kennedy-Krieger Institute and Johns Hopkins University; Kennedy-Krieger Institute and Johns Hopkins University

**Keywords:** down syndrome, IVIg, regression, neuropsychiatric, immunotherapy, neuroinflammation

## Abstract

Down syndrome regression disorder (DSRD) is a clinical symptom cluster consisting of neuropsychiatric regression without an identifiable cause. This study evaluated the clinical effectiveness of IVIg and evaluated clinical characteristics associated with relapse after therapy discontinuation. A prospective, multi-center, non-randomized, observational study was performed. Patients met criteria for DSRD and were treated with IVIg. All patients underwent a standardized wean off therapy after 9–12 months of treatment. Baseline, on therapy, and relapse scores of the Neuropsychiatric Inventory Total Score (NPITS), Clinical Global Impression-Severity (CGI-S), and the Bush-Francis Catatonia Rating Scale (BFCRS) were used to track clinical symptoms. Eighty-two individuals were enrolled in this study. Patients had lower BFCRS (MD: −6.68; 95% CI: −8.23, −5.14), CGI-S (MD: −1.27; 95% CI: −1.73, −0.81), and NPITS scores (MD: −6.50; 95% CI: −7.53, −5.47) while they were on therapy compared to baseline. Approximately 46% of the patients (n = 38) experienced neurologic relapse with wean of IVIg. Patients with neurologic relapse were more likely to have any abnormal neurodiagnostic study (χ^2^ = 11.82, p = 0.001), abnormal MRI (χ^2^ = 7.78, p = 0.005), and abnormal LP (χ^2^ = 5.45, p = 0.02), and a personal history of autoimmunity (OR: 6.11, p < 0.001) compared to patients without relapse. IVIg was highly effective in the treatment of DSRD. Individuals with a history of personal autoimmunity or neurodiagnostic abnormalities were more likely to relapse following weaning of immunotherapy, indicating the potential for, a chronic autoimmune etiology in some cases of DSRD.

## Introduction

Down syndrome (DS) is the most common cause of intellectual disability worldwide and occurs in 1 in 800 live births in the United States^1^. Neurologic and psychiatric diseases in this population are well described, although the last decade has seen an increasing frequency of reports of the onset of subacute developmental regression of unclear etiology in individuals considered too young to develop Alzheimer’s disease and too old to develop autism spectrum disorder. This condition has been referred to as Down Syndrome Regression Disorder (DSRD) and has primarily been reported in young persons with DS between ages 10 and 30 years^2,3^. Symptoms include a subacute loss of previously acquired developmental skills in the areas of language, communication, cognition, executive function, behavioral, and adaptive skills^2,4−7^. Other symptoms can include psychiatric manifestations, bradykinesia, catatonia, and rapid onset insomnia^4,5,7,8^. DSRD can be severe and significantly impact both the quality of life and autonomy of persons with DS.

Therapeutic interventions for this condition are broad and have ranged from antipsychotics to immunotherapy^4,9^. A minority of individuals with DSRD may have a neuroinflammatory etiology to the disease, confirmed by the presence of abnormal neurodiagnostic studies and dramatic immunotherapy responsiveness in some patients^4,9,10^. While immunotherapy provides a tool to rapidly reverse this clinical syndrome, guidance on dosing and duration of therapy remains unclear.

This study sought to examine changes in clinical measures of functionality, gait, catatonia, and neuropsychiatric symptoms among individuals with DSRD receiving IVIg, investigate possible demographic, lab, and clinical factors linked to responsiveness to immunotherapy with IVIg, and assess the likelihood of successful treatment tapering once improvement of symptoms has been achieved.

## Materials And Methods

### IRB and Data Availability

IRB approval was obtained for this study with waived assent authorized in patients not capable of providing assent. Consent was obtained from caregivers (if < 18 years) or legal guardians (if > 18 years) when assent could not be obtained. Anonymized data is available to qualified researchers upon request.

### Participants and Study Design

All individuals evaluated in the DS neurology clinic at multiple institutions were evaluated for participation in this study. Inclusion criteria included age between 8 and 26 years at the time of symptom onset, diagnosis of either possible or probable DSRD per expert consensus guidelines^3^, and completion of clinical neurodiagnostic studies (EEG, MRI, and Lumbar Puncture (LP)). Confirmation of diagnosis was performed by an arbiter with no knowledge of the case (MK). Exclusion criteria which included, age < 8 or > 26 years at the time of symptom onset, active cardiac or pulmonary disease, frequent infection (defined as more than two infections requiring antibiotics or antivirals per year), a history of neoplasia or receipt of chemotherapy, structural brain malformation on neuroimaging, active or a history of epilepsy (excluding febrile seizure), current use of electroconvulsive therapy and use of any immunotherapy not related to DSRD. Previously published cases of individuals receiving immunotherapy were also excluded^4,9^. Patients were permitted to be on psychotropic medications (e.g., selective serotonin reuptake inhibitor, antipsychotics, etc.) although once started on immunotherapy, dosing was locked with the exception of weaning if indicated. Individuals with co-morbid diagnoses of ASD were not excluded although they were all required meeting consensus criteria for DSRD.

Demographic data, medical history, and results of clinical and diagnostic investigations were collected through clinical documentation. Radiographic data was reviewed independently by a board-certified neuroradiologist. This study did not involve a control population of children with DS without DSRD as there are no other established indications for IVIg in DS.

### Visit Schedule

Prior to enrollment in the study, all patients were clinically evaluated and diagnosed with DSRD as per published guidelines (baseline)^3^. Patients were evaluated clinically at + 0 days (baseline), + 90 days, and + 180 days (+/− 7 days) after the initiation of IVIg. In addition to these scheduled visits, patients had the option for more frequent urgent visits when necessary. Behavioral and neuropsychiatric testing assessments were performed at baseline and at + 180 days. During the titration period, patients were evaluated at standardized time points of + 35 days, + 77 days, and + 119 days at the time of subsequent infusions (+/− 7 days). Behavioral and neuropsychiatric assessments were performed at the time of clinical relapse (urgent evaluation) or + 119 days, whichever came first.

### Behavioral/Neuropsychiatric Assessment

Given that DSRD has a wide variety of presenting symptoms, we employed several validated study tools in tandem to capture differences in disease severity. Research coordinators administered the Clinical Global Impression-Severity (CGI-S) scale at all clinical visits. At the baseline visit, the severity scale was performed and during follow up visits the improvement scale versions of this 7-point scale were administered. Physician evaluators also completed the Neuropsychiatric Inventory Questionnaire (NPI-Q) and the Bush-Francis Catatonia Rating Scale (BFCRS) at all clinical visits. These tests were utilized to capture global functional improvement (CGI), neuropsychiatric symptoms (NPI-Q), and catatonia (BFCRS). To assess global motor impairment, patients also had a timed 25-foot walk (25FTW) completed as part of their clinical evaluations when able to participate and follow directions. Higher scores for each of these metrics (CGI-S, NPI-Q, BFCRS, and 25FTW) was indicative of increased severity of symptoms.

### Definitions of Abnormal Neurodiagnostics

#### Electroencephalogram (EEG)

Focal or generalized slowing, focal epileptiform discharges out of any cortex, or seizure were considered abnormal. Generalized discharges were considered abnormal although inconsistent with the diagnosis of DSRD. All patients had to have at least one prior EEG that did not demonstrate these results previously.

#### MRI

All MRIs had to be performed on a 3T scanner with and without contrast administration. Any abnormality beyond a structural malformation (e.g., Chiari malformation) was considered abnormal. Patients did not require a prior “normal” MRI.

##### Lumbar Puncture (LP):

Abnormalities were defined as having any of the following: WBC count > 5 cells/mm3, total protein > 60 mg/dL, presence of oligoclonal bands, an IgG index of > 0.66, and/or an elevated neopterin (> 33 nmol/mL). Samples with over 1,000 RBC were excluded from analysis. Patients did not require a prior “normal” LP

### Therapeutic Interventions

A high concentration formulation of IVIg was utilized in all patients (10%, 100 mg/dL) at each dosing period. Patients were administered either Gammagard, Privagen, or Octagam formulations of IVIg depending on local infusion policies and regional restrictions on use. Once a patient was started on a particular formulation of IVIg, they had to continue on that same formulation unless an infusion reaction occurred. IVIg was dosed at 2 g/kg (administered over two days) for the induction dose followed by 1 g/kg (administered over one day) for maintenance dosing as per prior dosing regimens in pediatric inflammatory neurologic disease^11,12^. The timeframe between maintenance doses was 28 days +/−3 days and infusion protocols are presented in **Appendix A**. Steroids were not co-administered for any infusion unless as a treatment for medication reaction. Infusions could be administered at an outpatient infusion center or at home. In all situations, infusions were administered by a registered nurse.

### Therapeutic Wean

All patients were weaned off IVIg using a standard protocol. After 9 months of IVIg therapy, the frequency of infusions was reduced from every four to every five weeks, then to every six weeks, then to every seven. Completed wean off therapy would take 18 total weeks. If there was no clinical return of symptoms (e.g., catatonia, mutism etc.), IVIg therapy was then discontinued; if there was recurrence of symptoms, the patient was placed back on an every four week infusion schedule. A relapse was defined as any sustained worsening (≥ 3 days) in any of the symptoms listed on the international DSRD criteria checklist and was determined by the evaluating clinician^3^.

### Safety Assessments

Patients were asked standardized screening questions to report any adverse events (AE) on therapy at two time points (+ 90 days and + 180 days (+/− 7 days)) during the study period; they were also asked about intercurrent use of antibiotics or antivirals, urgent or emergent medical care evaluations, hospitalizations, or febrile illnesses. All potential AEs were reported.

During infusions, a nurse was available at bedside for rapid triage of reactions to IVIg administration. All infusion reactions were evaluated and escalated to the treating physician when appropriate. At the first sign of an infusion reaction, the infusion was paused, and only resumed after medical clearance by the supervising physician.

### Statistical Analysis

The primary outcomes of interest were the 25FTW, BFCRS, CGI-S, NPI-Q Total Scores (NPITS) collected at multiple time points (i.e., baseline, on-therapy, and after-therapy). These scores were analyzed using mixed-effects regression models with an unstructured covariance model for the “within subject repeated measures” and “indicators for time post-baseline to capture change in outcome mean scores from baseline”. The models further allowed for fixed effects for demographics and disease biometrics and their interactions with time, with effects added individually.

Additional mixed-effects models with similar covariance structure were used including the fixed effects for those with relapse relative to those without, categorical indicators for time post-baseline, and the interaction between relapse and time. The models further allowed for interaction effects between demographics and disease biometrics and relapse and time.

Demographics, disease biometrics, and baseline clinical features were compared between patients with and without neurologic relapse using chi-square (χ^2^) or Fisher’s exact tests for categorical variables, and *t*-test for continuous variables. Univariate logistic regression was used to model the association between relapse and individual demographics, disease biometrics, and baseline clinical features. Factors which differed significantly by relapse were entered into a separate multivariable logistic regression model. All analyses were conducted using Stata/MP version-17.0 (StataCorp. 2021. College Station, TX: StataCorp LLC.).

## Results

### Demographics and Clinical Features

Ninety-three patients were identified for potential review, of which 82 (88%) met all inclusion criteria. The most common reasons for exclusions were age > 26 years at symptom onset (n = 8, 73%), prior receipt of immunotherapy unrelated to DSRD (n = 2, 18%), and history of epilepsy (n = 1, 9%). Demographics and clinical features of cases are reported in Table 1.

### Therapeutics and Safety

Gammagard was the most common IVIg formulation administered (67%). In total, only two patients (2.4%) had AEs reported during the study period. One patient had an infusion reaction (rash) during the third infusion and one developed wheezing two hours into her fourth infusion. Infusions were temporarily paused but completed after therapeutic intervention. Both patients continued to receive infusions with no further AEs. No participant developed deep venous thrombosis or clotting, headache, aseptic meningitis, or other known side effects of IVIg. Nearly 20% (n = 16) of patient’s caregivers reported subjective improvements in skin conditions such as hidradenitis suppurativa, eczema, and psoriasis. This was not systematically asked by clinicians but was information volunteered by families in some circumstances.

### Therapeutic Effects

**Figure 1:**
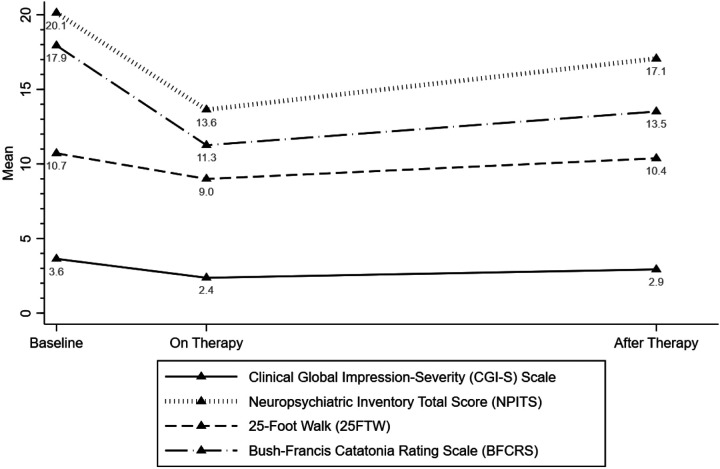
Clinical features including behavioral and neuropsychiatric assessments over the study period.

Changes in primary clinical outcome measures are displayed in Fig. 1 and Table 2. While on therapy, in comparison to baseline, patients had lower scores for 25FTW (mean difference (MD): −1.72; 95% confidence interval (CI): −2.42, −1.01), BFCRS (MD: −6.68; 95% CI: −8.23, −5.14), CGI-S (MD: −1.27; 95% CI: −1.73, −0.81), and NPITS (MD: −6.50; 95% CI: −7.53, −5.47). Furthermore, after therapy, lower mean scores were observed for BFCRS (MD: −4.43; 95% CI: −5.89, −2.97), CGI-S (MD: −0.71; 95% CI: −0.95, −0.47), and NPITS (MD: −3.07; 95% CI: −3.91, −2.23) but not 25FTW (MD: −0.34; 95% CI: −0.91, 0.24), compared to baseline.

### Clinical Response Variables:

There was evidence that changes in clinical responses differed by disease-related factors such as the presence of catatonia and treatment with prior immunotherapy as well as neurodiagnostic study abnormalities. Clinical responses were more profound in individuals with catatonia, those who had received prior immunotherapy for the treatment of DSRD and those with any neurodiagnostic study abnormality (Table 3).

In total, there were 12 individuals who did not respond to therapy. Amongst non-responders, 11/12 (93%) had no neurodiagnostic study abnormalities, 11/12 (93%) had no altered mental status, 11/12 (93%) had no developmental regression (7%), and 10/12 (23%) had no catatonia. Additionally, 8/12 (67%) had neither altered mental status, developmental regression or catatonia.

### Neurodiagnostic Abnormalities and Disease Severity

Patients with any neurodiagnostic abnormalities had higher means at baseline for 25FTW (MD: 3.50; 95% CI: 0.84, 6.15) and Total NPI (MD: 3.16; 95% CI: 0.42, 5.90) compared to those without any abnormalities. Lower mean of CGI-S (MD: −0.81; 95% CI: −1.26, −0.37) was observed for patients with any neurodiagnostic abnormalities while on-therapy compared to baseline. Moreover, patients with any neurodiagnostic abnormalities had higher mean scores of 25FTW (MD: 4.67; 95% CI: 2.70, 7.07), BFCRS (MD: 6.69; 95% CI:2.40, 10.98), CGI-S (MD:1.13; 95% CI: 0.43, 1.84), and Total NPI (MD: 5.17; 95% CI: 2.51, 7.83) after-therapy compared to those without any abnormalities. A borderline difference was observed between patients with and without neurodiagnostic abnormalities while on-therapy (MD: 1.35; 95% CI: −0.002, 2.71).

With regards to specific neurodiagnostics, patients with EEG abnormality had lower mean for BFCRS and NPITS while on-therapy and after-therapy relative to baseline. Similar pattern was observed for patients without EEG abnormality while on-therapy and after-therapy compared to baseline. Patients with abnormal EEG had lower mean NPITS while on-therapy (MD: −3.54; 95% CI: −6.04, −1.05) compared to those without EEG abnormality. Patients with abnormal neuroimaging had lower means for BFCRS, CGI-S, and NPITS while on-therapy compared to baseline. Similarly, patients without neuroimaging abnormalities had lower BFCRS, CGI-S, and NPITS while on-therapy and also after-therapy compared to baseline. While patients with abnormal neuroimaging had lower mean for CGI-S while on-therapy (MD: −0.69; 95% CI: −1.24, −0.14) compared to those with normal neuroimaging, they had higher means of CGI-S (MD: 1.28; 95% CI: 0.44, 2.12) and NPITS (MD: 5.00; 95% CI: 1.76, 8.24) after-therapy compared to patients with normal neuroimaging.

Patients with LP abnormalities had lower means of BFCRS, CGI-S, and NPITS while-on therapy compared to baseline. Those with a normal LP had lower mean levels of BFCRS, CGI-S, and NPITS while on-therapy and after-therapy relative to baseline. Patients with abnormal LP had higher mean levels at baseline for CGI-S (MD: 0.93; 95% CI: 0.04, 1.83) and NPITS (MD: 3.88; 95% CI: 0.26, 7.51) compared with patients without such abnormality. Moreover, higher mean levels for CGI-S (MD: 1.47; 95% CI: 0.53, 2.40) and NPITS (MD: 5.77; 95% CI: 2.17, 9.36) were observed for patients with LP abnormality after-therapy compared to those without abnormality.

### Change in Clinical Features Incorporating Neurologic Relapse

**Figure 2:**
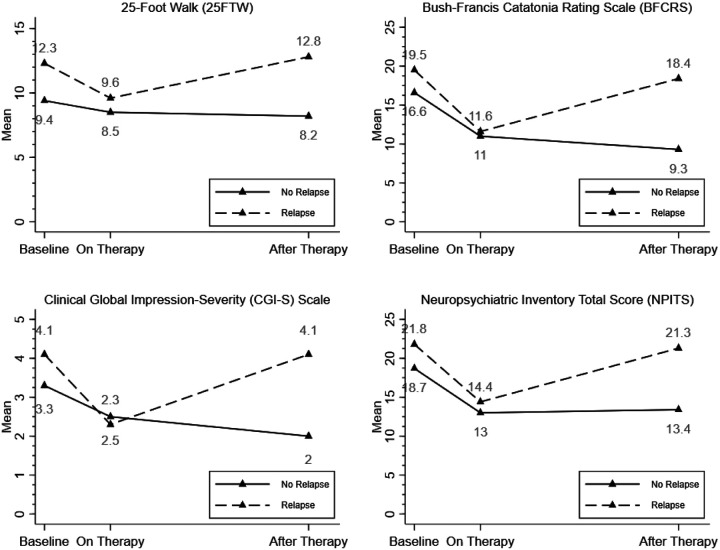
Clinical features including behavioral and neuropsychiatric assessments over the study period for patients with and without neurologic relapse.

Therapeutic response across all clinical measures was sustained in individuals who did not relapse although those that did relapse had scores return to baseline levels (Fig. 2). We observed a significant reduction in mean scores of all clinical outcomes while on-therapy compared to baseline for patients with relapse (**Appendix B**). For patients without relapse, there was also evidence of reduction in means of all the outcomes while on-therapy compared to baseline, except for 25FTW. Additionally, significant reduction in scores were observed among these patients without relapse when comparing after-therapy with baseline. Patients with relapse had higher baseline means for all the clinical outcomes, except for BFCRS and higher mean scores for all the clinical outcomes after-therapy compared to patients without relapse (**Appendix C**)

### Clinical Features and Relapse

There was evidence that the association between clinical responses and sex, catatonia, any neurodiagnostic abnormalities, and treatment with prior immunotherapy differed by neurologic relapse. Of note, individuals with any neurodiagnostic abnormality who did relapse had lower mean scores on the 25FTW while on-therapy and higher mean after-therapy relative to baseline. Sub analysis of the impact of non-IVIg medications (e.g., anti-depressants) on relapse was not possible due to highly heterogenous treatments, yielding only 12 patients with identical regimens.

### Risk of Relapse and Neurodiagnostic Abnormalities

**Figure 3:**
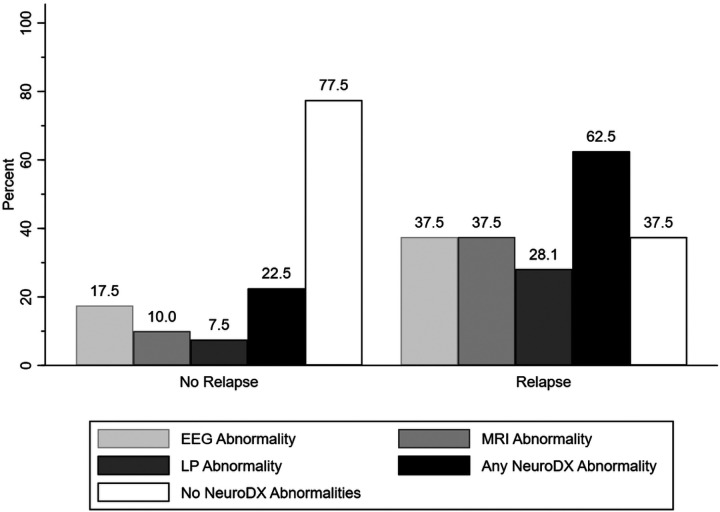
Neurodiagnostic abnormality presence in patients with (n = 44) and without (n = 38) neurologic relapse.

Approximately 46% of the patients (n = 38) experienced a relapse of symptoms. Patients who relapsed were more likely to have any abnormal neurodiagnostic study (χ^2^ = 11.82; p = 0.001), abnormal MRI (χ^2^ = 7.78; p = 0.005), and abnormal LP (χ^2^ = 5.45; p = 0.02) compared to patients without relapse (Fig. 3). Individuals with a history of personal autoimmunity were six times more likely to relapse than those without (OR: 6.11, p < 0.001, 95%CI: 2.69–12.13). Additionally, patients with relapse had higher baseline 25FTW (p = 0.0222), CGI Severity of illness Score (p = 0.0152), NPI Irritability Score (p = 0.005), and NPITS (p = 0.0171) than those without relapse (Table 1).

### Predictors of Relapse

#### Unadjusted analysis

There was no evidence of significant association between individual demographic characteristics and neurologic relapse after therapy, except a borderline association with the difference between age at therapy and age at diagnosis (OR: 2.20, 95% CI: 0.98, 4.93). Higher odds of relapse were associated with MRI abnormality (OR: 5.40, 95% CI: 1.54, 18.97), LP abnormality (OR: 4.83, 95% CI: 1.18, 19.70), and any neurodiagnostic abnormality (OR: 5.74, 95% CI: 2.05, 16.10).

Relapse was associated with a number of baseline clinical features, where the odds of relapse increased by one unit for each increase in 25FTW (OR: 1.11, 95% CI: 1.01, 1.21), CGI-S (OR: 1.49, 95% CI: 1.07, 2.07), NPI irritability (OR: 1.65, 95% CI: 1.14, 2.39), and NPITS (OR: 1.10, 95% CI: 1.01, 1.19). In addition, borderline associations were observed between relapse and baseline NPI Hallucinations (OR: 1.31, 95% CI: 0.97, 1.77) and NPI Apathy (OR: 1.32, 95% CI: 0.98, 1.77) scores.

#### Adjusted analysis

The disease biometrics and baseline clinical features that differed significantly by relapse were included in a model adjusting for sex, ethnicity, and difference between age at therapy and age at diagnosis. Analysis revealed a greater risk of relapse after therapy was associated with any neurodiagnostic abnormality (aOR: 4.34, 95% CI: 1.39, 13.53). No evidence of significant association was observed between relapse and other baseline covariates was present.

## Discussion

Individuals with DSRD were responsive to immunotherapy on a variety of clinical measures, consistent with previously published data^4,9,10^. Patients demonstrated improvements in functional status (CGI), gait (25FTW), catatonia (BFCRS and 25FTW), and neuropsychiatric symptoms (NPITS). Further, treatment of these patients for a period of 9–12 months yielded sustained improvement in 47% of individuals after IVIg immunotherapy was weaned off. Those with a history of personal autoimmunity or baseline neurodiagnostic abnormalities were more likely to experience a clinical relapse upon wean of immunotherapy. These findings advance our understanding of the DSRD phenotype having immunologic origins in a subset of individuals.

Beneficial clinical responses to immunotherapy (IVIg) were high in this cohort at roughly 85% (70/82), consistent with prior studies^4,9,10^. These improvements were most notable in individuals with catatonia or neurodiagnostic study abnormalities, which is consistent with previously published data^4^. Non-responders to therapy were less likely to have neurodiagnostic study abnormalities (7%), altered mental status (7%), developmental regression (7%), and catatonia (17%). Although these findings were observed in a limited cohort (n = 12), it does indicate that some clinical features could be more predictive of non-immunotherapy responsive disease. Conversely, this study also expands on the concept that even in patients without definitive neurodiagnostic abnormalities, there may be a role for immune-based interventions as well, highlighting need to prioritize identification of sensitive and specific biomarkers in DSRD.

Duration of immunotherapy has remained an important question in individuals with DSRD since the first cohorts of immunotherapy-responsive patients were published.^10^ This study used a slow therapeutic wean, consistent with previously published literature, in order to avoid rebound after abrupt discontinuation^13,14^. Ultimately, the 53% of individuals that did relapse upon wean of IVIg may represent a cohort of individuals where the etiology of their DSRD-related symptoms is potentially inflammatory in nature. Etiologies to DSRD remain poorly elucidated although emerging evidence for a neuroinflammatory component to the disease in a minority of individuals has gained traction^3,9^. Thus, the data presented in this report not only serves to highlight the therapeutic effect of IVIg in DSRD, but also provides proof of concept that the immunomodulatory effects of this therapeutic may be treating a potential, albeit unknown, inflammatory target. This is supported the observed 4.34 times greater risk of relapse in individuals with neurodiagnostic study abnormalities (including early/accelerated mineralization and CSF abnormalities) which are indicative of potentially interferon driven immune dysregulation, a concept more well established in systemic disease presentations in persons with DS^15–18^. It has been established that individuals with DS are at great risk for a variety of autoimmune disorders^19–25^, and thus it would be reasonable to consider the brain as another potential target of immune dysregulation.

This study is not without limitations. Selection and severity bias is present in the exclusion of patients who did not undergo neurodiagnostic work up. Investigators involved in this study were early adopters of immunotherapy in persons with DSRD and thus both a referral and selection bias in favor of use of IVIg may be present. Severity bias could also decrease the likelihood of response to any therapy, but the authors do contemplate if more severe cases are associated with inflammatory etiologies which is still undetermined. This same severity bias could have explained the responsiveness to IVIg and the high rates of neurologic relapse in this data set. Sub analysis of non-immunotherapy was not possible in this study due to the low numbers of patients on the same regimen. Clinical treatment heterogeneity, utilization of weight-based dosing and lack of algorithmic therapeutic interventions in DSRD are major limiting factors in this disease and yielded only 12 patients who were on exactly the same treatments at the time of immunotherapy initiation. In addition, patients were on a variety of different psychotropic medications at baseline and while these were not changed during this study, add an additional layer of complexity to interpreting and generalizing the results. With regards to metrics, the use of CGI is subjective measure prone to recall bias, although this was mitigated by the use of two objective, physician-based metrics. This study did not assess family response to treatments (e.g., functional improvements in homelife for family) although the authors acknowledge this would be an important variable to assess in future study. Importantly, this study was not randomized nor controlled which should temper interpretation. Standardized immunotherapy regimens and wean schedules mitigated some of this although further randomized, controlled, trials are desperately needed in this space. Different formulations of IVIg had to be used although no significant differences between formulations was identified. Additionally in our demographic and clinic response variable analysis, we observed statistical (and lack thereof) differences in study tools; this may reflect differences in disease severity between those comparison groups or more likely reflect the study’s low power. Finally, the authors note that given the rare nature of DSRD, low study populations limit the generalizability of these findings and the authors caution clinicians to evaluate each case individually.

In summary, amongst a cohort of individuals with DSRD, immunotherapy was effective in the treatment of the clinical symptom cluster. Individuals with neurodiagnostic abnormalities of any type were significantly less likely to be able to wean off of immunotherapy, indicating the potential for, a chronic immune etiology in some cases of DSRD. These results must be tempered by multiple study limitations although provide a basis for further investigations into randomized controlled, double-blinded, biomarker and therapeutic trials in this emerging disease.

## Figures and Tables

**Table 1:** Demographics and clinical data (N = 82)

	No Relapse (n = 44; 53.7%)		Relapse (n = 38; 46.3%)		All (n = 82)	
Demographics						
Sex						
Female	26	59.1%	18	47.4%	44	53.7%
Male	18	40.9%	20	52.6%	38	46.3%
Race						
Caucasian	34	77.3%	31	81.6%	65	79.3%
Black	6	13.6%	4	10.5%	10	12.2%
Asian	4	9.1%	3	7.9%	7	8.5%
Ethnicity						
Non-Hispanic	13	29.5%	13	34.2%	26	31.7%
Hispanic	31	70.5%	25	65.8%	56	68.3%
Age at symptom onset	14.1	(3.5)	15.6	(4.6)	14.8	(4.1)
Age at diagnosis	16.5	(4.1)	17.9	(5.1)	17.2	(4.6)
Age at Therapy	16.8	(4.1)	18.4	(4.9)	17.5	(4.5)
Δ Age at Symptom Onset and Age at Diagnosis	2.4	(1.9)	2.3	(1.8)	2.4	(1.8)
Δ Age at Therapy and Age at Diagnosis*	0.3	(0.5)	0.5	(0.6)	0.4	(0.6)
Δ Age at Therapy and Age at Symptom Onset	2.7	(2.1)	2.8	(1.7)	2.8	(1.9)
Time (months) to Symptom Peak	3.7	(2.5)	3.3	(2.5)	3.5	(2.5)
Trigger Present	20	45.5%	20	52.6%	40	48.8%
Type of Trigger						
Infection	9	20.5%	7	18.4%	16	19.5%
Change of School/Work/Home Environment	6	13.6%	5	13.2%	11	13.4%
Loss of Family/Caregiver/Friend	2	4.5%	2	5.3%	4	4.9%
Death	1	2.3%	2	5.3%	3	3.7%
Change in Residence	0	0.0%	2	5.3%	2	2.4%
Abuse	1	2.3%	1	2.6%	2	2.4%
Medical Change	1	2.3%	1	2.6%	2	2.4%
Disease Biometrics						
Probable DSRD Criteria	17	38.6%	13	34.2%	30	36.6%
History of Personal Autoimmune Disease	18	45.0%	15	46.9%	33	45.8%
Serum Cytokines	7	26.9%	13	54.2%	20	40.0%
Catatonia	30	68.2%	30	78.9%	60	73.2%
EEG Abnormal*	7	17.5%	12	37.5%	19	26.4%
MRI Abnormal**	4	10.0%	12	37.5%	16	22.2%
Lumbar Puncture Abnormal**	3	7.5%	9	28.1%	12	16.7%
Any Neurodiagnostic Study Abnormal**	9	22.5%	20	62.5%	29	40.3%
Psychotropic Medications at Baseline	35	79.5%	30	78.9%	65	79.3%
Benzodiazepines	22	50%	17	44.7%	39	47.6%
SSRI/SNRIs	17	38.6%	10	26.3%	27	32.9%
Antipsychotics	8	18.2%	7	18.4%	17	20.7%
Anticonvulsants	2	4.5%	5	13.1%	7	8.5%
Mood Stabilizers	3	6.8%	0	0%	3	3.6%
Prior Immunotherapy	3	6.8%	1	2.6%	4	4.9%
IVIg Brand						
Gammaguard	27	61.4%	28	73.7%	55	67.1%
Octagam	11	25.0%	7	18.4%	18	22.0%
Privagen	6	13.6%	3	7.9%	9	11.0%
IVIg Duration	7.5	(2.4)	7.8	(2.1)	7.6	(2.3)
Baseline Clinical Features						
25 Foot Walk**	9.4	(4.5)	12.3	(6.4)	10.7	(5.6)
Bush Francis Severity Score	16.6	(9.9)	19.5	(10.9)	17.9	(10.4)
CGI Severity of Illness**	3.3	(1.3)	4.1	(1.5)	3.6	(1.4)
NPI Total Score**	18.7	(5.7)	21.8	(5.9)	20.1	(6.0)
NPI Delusions	3.8	(1.9)	3.6	(1.6)	3.7	(1.8)
NPI Hallucinations*	1.2	(1.4)	1.8	(1.7)	1.5	(1.5)
NPI Agitation	2.1	(1.4)	1.9	(1.6)	2.0	(1.5)
NPI Anxiety	0.8	(0.9)	0.6	(0.7)	0.7	(0.8)
NPI Apathy*	2.5	(1.5)	3.1	(1.6)	2.8	(1.5)
NPI Irritability**	1.7	(1.2)	2.5	(1.5)	2.1	(1.4)
NPI Euphoria	0.5	(0.8)	0.6	(0.8)	0.5	(0.8)
NPI Disinhibition	0.5	(0.9)	0.7	(1.4)	0.5	(1.2)
NPI Aberrant Motor	2.6	(1.7)	3.3	(1.9)	2.9	(1.8)
NPI Night Time	2.3	(1.7)	2.7	(1.5)	2.5	(1.6)
NPI Appetite/Eating	0.7	(1.0)	1.0	(1.5)	0.9	(1.2)

* *p* < 0.1 (italic font);

** *p* < 0.05 (bold font).

Data are mean (SD) or frequency and percentage %. The frequency and percentages of incomplete variables are as follows: History of Personal Autoimmune Disease (n = 10; 12.2%), Serum Cytokines (n = 32; 39%), EEG Abnormal (n = 10; 12.2%), MRI Abnormal (n = 10; 12.2%), Lumbar Puncture Abnormal (n = 10; 12.2%), and Any Neurodiagnostic Study Abnormal (n = 10; 12.2%). Δ = difference between. Multiple responses allotted for types of trigger and psychotropic medications at baseline.

**Table 2:** Estimated regression coefficient with 95% confidence interval [CI] from repeated measures analyses for all patients.

	Coef.	SE	95% CI	p-value
25-Foot walk				
Prior to Therapy	0.00	0.00		
On-Therapy**	−1.72	0.36	[−2.42, −1.01]	0.0000
After-Therapy	−0.34	0.29	[−0.91, 0.24]	0.2509
Bush-Francis Score				
Prior to Therapy	0.00	0.00		
On-Therapy**	−6.68	0.79	[−8.23, −5.14]	0.0000
After-Therapy**	−4.43	0.75	[−5.89, −2.97]	0.0000
CGI-Severity Score				
Prior to Therapy	0.00	0.00		
On-Therapy**	−1.27	0.23	[−1.73, −0.81]	0.0000
After-Therapy**	−0.71	0.12	[−0.95, −0.47]	0.0000
Total NPI Score				
Prior to Therapy	0.00	0.00		
On-Therapy**	−6.50	0.53	[−7.53, −5.47]	0.0000
After-Therapy**	−3.07	0.43	[−3.91, −2.23]	0.0000

* *p* < 0.1 (italic font);

** *p* < 0.05 (bold font).

The repeated measure analyses were carried out using longitudinal mixed-effects regression models with restricted maximum likelihood estimation and Kenward-Roger method for small-sample adjustment

**Table 3:** Estimated means with 95% confidence interval [CI] for clinical outcomes differed by potential disease biometrics

	On-Therapy				After-Therapy			
	Mean change from baseline	SE	95% CI	p-value	Mean change from baseline	SE	95% CI	p-value
25-Foot walk								
With catatonia	−2.39	0.40	[−3.17, −1.62]	0.0000	−0.75	0.33	[−1.40, −0.09]	0.0254
Without catatonia	0.13	0.65	[−1.15, 1.41]	0.8457	0.78	0.55	[−0.30, 1.86]	0.1580
With any neurodiagnostic abnormalities	−3.14	0.60	[−4.32, −1.96]	0.0000	0.31	0.551	[−0.68, 1.30]	0.5397
Without any neurodiagnostic abnormalities	−1.00	0.50	[−1.97, −0.02]	0.0447	−0.86	0.42	[−1.68, −0.05]	0.0379
Prior immunotherapy	−4.05	1.61	[−7.21, −0.89]	0.0120	−4.07	1.26	[−6.55, −1.60]	0.0013
No prior immunotherapy	−1.60	0.37	[−2.31, −0.88]	0.0000	−0.14	0.29	[−0.71, 0.42]	0.6130
Bush-Francis Score								
With catatonia	−8.45	0.84	[−10.10, −6.80]	0.0000	−5.45	0.85	[−7.11, −3.79]	0.0000
Without catatonia	−1.86	1.39	[−4.59, 0.87]	0.1811	−1.64	1.40	[−4.38, 1.11]	0.2432
With any neurodiagnostic abnormalities	−9.86	1.30	[−12.41, −7.32]	0.0000	−3.45	1.29	[−5.98, −0.92]	0.0075
Without any neurodiagnostic abnormalities	−5.28	1.07	[−7.37, −3.19]	0.0000	−5.56	1.06	[−7.63, −3.48]	0.0000
EEG abnormal	−10.42	1.62	[−13.60, −7.24]	0.0000	−6.00	1.60	[−9.14, −2.86]	0.0002
EEG normal	−5.94	0.97	[−7.85, −4.04]	0.0000	−4.25	0.96	[−6.12, −2.37]	0.0000
MRI abnormal	−8.44	1.83	[−12.02, −4.85]	0.0000	−1.56	1.70	[−4.90, 1.78]	0.3590
MRI normal	−6.75	0.98	[−8.67, −4.83]	0.0000	−5.61	0.91	[−7.39, −3.82]	0.0000
LP abnormal	−9.42	1.41	[−12.17, −6.66]	0.0000	−1.67	1.15	[−3.92, 0.59]	0.1470
LP normal	−6.25	0.63	[−7.48, −5.02]	0.0000	−3.55	0.51	[−4.56, −2.54]	0.0000
Prior immunotherapy	−9.00	3.58	[−16.01, −1.99]	0.0119	−11.25	3.31	[−17.73, −4.77]	0.0007
No prior immunotherapy	−6.56	0.81	[−8.15, −4.98]	0.0000	−4.08	0.75	[−5.54, −2.61]	0.0000
CGI-Severity Score								
With catatonia	−1.58	0.27	[−2.10, −1.06]	0.0000	−0.63	0.14	[−0.92, −0.35]	0.0000
Without catatonia	−0.41	0.44	[−1.27, 0.45]	0.3519	−0.91	0.24	[−1.38, −0.44]	0.0001
With any neurodiagnostic abnormalities	−2.21	0.38	[−2.96, −1.46]	0.0000	−0.45	0.21	[−0.86, −0.04]	0.0324
Without any neurodiagnostic abnormalities	−0.81	0.31	[−1.43, −0.20]	0.0096	−1.00	0.17	[−1.34, −0.66]	0.0000
MRI abnormal	−2.31	0.53	[−3.35, −1.28]	0.0000	−0.19	0.28	[−0.73, 0.36]	0.5018
MRI normal	−1.11	0.28	[−1.66, −0.55]	0.0001	−0.95	0.15	[−1.24, −0.65]	0.0000
LP abnormal	−2.58	0.61	[−3.77, −1.39]	0.0000	−0.33	0.33	[−0.98, 0.31]	0.3128
LP normal	−1.13	0.27	[−1.67, −0.60]	0.0000	−0.87	0.15	[−1.16, −0.58]	0.0000
Total NPI Score								
With catatonia	−7.40	0.59	[−8.55, −6.25]	0.0000	−3.22	0.50	[−4.20, −2.23]	0.0000
Without catatonia	−4.05	0.97	[−5.95, −2.14]	0.0000	−2.68	0.83	[−4.31, −1.05]	0.0012
With any neurodiagnostic abnormalities	−9.55	0.83	[−11.17, −7.93]	0.0000	−2.03	0.73	[−3.46, −0.61]	0.0052
Without any neurodiagnostic abnormalities	−4.91	0.68	[−6.24, −3.58]	0.0000	−4.05	0.60	[−5.22, −2.88]	0.0000
EEG abnormal	−10.00	1.06	[−12.08, −7.92]	0.0000	−2.11	0.91	[−3.90, −0.31]	0.0213
EEG normal	−5.62	0.63	[−6.87, −4.38]	0.0000	−3.64	0.55	[−4.71, −2.57]	0.0000
MRI abnormal	−10.13	1.17	[−12.42, −7.83]	0.0000	−1.81	0.99	[−3.76, 0.13]	0.0678
MRI normal	−5.82	0.62	[−7.05, −4.60]	0.0000	−3.64	0.53	[−4.68, −2.60]	0.0000
LP abnormal	−9.42	1.41	[−12.17, −6.66]	0.0000	−1.67	1.15	[−3.92, 0.59]	0.1470
LP normal	−6.25	0.63	[−7.48, −5.02]	0.0000	−3.55	0.51	[−4.56, −2.54]	0.0000

*p* < 0.1 (italic font); *p* < 0.05 (bold font).

## Abbreviations

25FTW: Timed 25-foot walk
95% CI: 95% Confidence Interval
BFCRS: Bush-Francis Catatonia Rating Scale
CGI-S: Clinical Global Impression-Severity Score
CSF: Cerebrospinal Fluid
DS: Down Syndrome
DSRD: Down Syndrome Regression Disorder
EEG: Electroencephalogram
IVIg: Intravenous Immunoglobulin
LP: Lumbar puncture
MD: Mean Difference
MRI: Magnetic Resonance Imaging
NPITS: Neuropsychiatric Inventory Total Score
